# Local but not long-range microstructural differences of the ventral temporal cortex in developmental prosopagnosia

**DOI:** 10.1016/j.neuropsychologia.2015.10.010

**Published:** 2015-11

**Authors:** Sunbin Song, Lúcia Garrido, Zoltan Nagy, Siawoosh Mohammadi, Adam Steel, Jon Driver, Ray J. Dolan, Bradley Duchaine, Nicholas Furl

**Affiliations:** a Human Cortical Physiology Section, National Institute of Neurological Disorders and Stroke, National Institutes of Health, Bethesda, MD 20892, USA; b Division of Psychology, Department of Life Sciences, Brunel University, Uxbridge UB8 3PH, United Kingdom; c Laboratory for Social and Neural Systems Research (SNS Lab), University of Zurich, Rämistr. 100, CH-8091 Zurich, Switzerland; d Wellcome Trust Centre for Neuroimaging, University College London, London WC1N 3BG, United Kingdom; e Department of Systems Neuroscience, University Medical Center Hamburg-Eppendorf, Hamburg, Germany; f Institute of Cognitive Neuroscience, University College London, London WC1N 3AR, United Kingdom; g Psychological and Brain Sciences, Dartmouth College, Hanover, NH 03755, USA; h Department of Psychology, Royal Holloway, University of London, Egham Hill, Egham, Surrey TW20 0EX, United Kingdom

**Keywords:** Diffusion-weighted imaging, Inferior longitudinal fasciculus, Inferior fronto-occipital fasciculus, Prosopagnosia, Face perception, Individual differences

## Abstract

Individuals with developmental prosopagnosia (DP) experience face recognition impairments despite normal intellect and low-level vision and no history of brain damage. Prior studies using diffusion tensor imaging in small samples of subjects with DP (*n*=6 or *n*=8) offer conflicting views on the neurobiological bases for DP, with one suggesting white matter differences in two major long-range tracts running through the temporal cortex, and another suggesting white matter differences confined to fibers local to ventral temporal face-specific functional regions of interest (fROIs) in the fusiform gyrus. Here, we address these inconsistent findings using a comprehensive set of analyzes in a sample of DP subjects larger than both prior studies combined (*n*=16). While we found no microstructural differences in long-range tracts between DP and age-matched control participants, we found differences local to face-specific fROIs, and relationships between these microstructural measures with face recognition ability. We conclude that subtle differences in local rather than long-range tracts in the ventral temporal lobe are more likely associated with developmental prosopagnosia.

## Highlights

• Developmental prosopagnosics (*n*=16) show white matter deficits local to FFA.
• Fractional anisotropy in WM local to FFA correlates with face identity recognition.
• Contrary to prior reports, long-range tracts (IFOF and ILF) in DP appear normal.
• Findings are additional evidence that DP often involves posterior regions.

## 1 Introduction

People with prosopagnosia experience severe deficits with facial identity recognition despite normal low-level vision and normal intellect. Prosopagnosia can occur due to a failure to develop the mechanisms necessary for face recognition, and when it does so in the absence of more general neurodevelopmental disorders, it is referred to as developmental prosopagnosia (DP) or congenital prosopagnosia (Susilo and Duchaine, 2013, Behrmann and Avidan, 2005a, Behrmann et al., 2005b). Rough estimates suggest that the prevalence of DP is about 2% (Kennerknecht et al., 2006, Kennerknecht et al., 2008). Not surprisingly, the social difficulties DP creates lead to elevated rates of psychosocial problems (Dalrymple et al., 2014a, Yardley et al., 2008).

Face recognition depends on a network of spatially distributed regions in the occipital and temporal cortices, and proper functioning of this network depends on the structural connections between these regions. A study by Thomas et al. (2009) implicated impaired microstructural integrity of the two major long-range tracts projecting from posterior occipito-temporal regions to anterior temporal and frontal lobe regions (the inferior longitudinal fasciculus (ILF) and the inferior fronto-occipital fasciculus (IFOF) respectively) as a critical neural feature of DP. That study used diffusion tensor imaging (DTI) and deterministic tractography and found that, relative to a group of controls, six DP participants showed reductions in the integrity of the ILF and the IFOF bilaterally as assessed by mean fractional anisotropy (FA), numbers of fibers, and tract volume. In combination with functional MRI studies showing normal activity in posterior face-selective regions (Avidan et al., 2005, Avidan et al., 2011, Hasson et al., 2003), these structural deficits were interpreted as evidence for DP as a disconnection syndrome: face processing deficits occur because intact posterior occipito-temporal regions that are responsible for visual analysis of faces are unable to communicate via the ILF and IFOF with more anterior temporal areas (Avidan and Behrmann, 2009; Avidan et al., 2014; Behrmann and Plaut, 2013).

However a more recent paper did not find any group differences between DP and control subjects in the ILF (they did not analyze the IFOF) (Gomez et al., 2015). This study compared eight subjects with DP to controls and instead found more localized differences within fibers defined by tractography from face-specific functional regions of interest located within a region in the fusiform gyrus (Gomez et al., 2015) known as the fusiform face area (FFA).

The study by Thomas et al. (2009), conducted during the early days of diffusion tensor imaging, employed limited scanning parameters for diffusion data (6 diffusion directions), that are now considered less than ideal for tractography (Berman et al., 2013, Thomas et al., 2014). Further, while both studies based much of their findings on tractography-based metrics, recent studies have demonstrated the substantial influence of different tracking algorithms on tracts identified, and called into question the ability of any tracking algorithm to be both sensitive and specific (Thomas et al., 2014), or able to differentiate superficial white matter fiber systems from long-range connections (Reveley et al., 2015). These studies point out the inherent limitations of tractography methods to distinguish between tracts.

For these reasons, we made the following substantial improvements in data collection and additions to data analyzes. We used scanning parameters for diffusion data (two datasets with 61 diffusion directions each) and corrections for susceptibility-induced image distortions (Andersson et al., 2003) that allows for more precise, reliable, and accurate tractography as well as better estimation of FA (Wang et al., 2012, Jones, 2011). We included a more thorough set of blinded analyzes that, defined tracts deterministically with varied curvature thresholds as well as probabilistically. Given the inherent limitations of tracting algorithms to differentiate between tracts, we also included voxel-wise comparisons within a mask that included all tracts and fibers of interest, given that voxel-wise comparisons do not rely on the accuracy of tractography. However, given the introduction of Type 1 errors with the problem of multiple voxel-wise comparisons, we used Monte-Carlo simulations to determine family-wise error to qualify findings. We additionally tested whole brain voxel-wise comparisons like those employed by Thomas et al. (2009) though that report did not highlight family-wise error as we do here. The problem of multiple comparisons increases dramatically with a whole brain search (Supplementary Section 1).

Finally, as pointed out by both Thomas et al. (2009) and Gomez et al. (2015), the small numbers of subjects included in those studies (*n*=6 and *n*=8) required validation in larger numbers of subjects. Here, we address past inconsistent findings in a cohort of subjects with DP that is larger than both prior DTI studies combined (*n*=16), with the added benefit that these subjects have been well characterized behaviorally (Dalrymple et al., 2014b; Garrido et al., 2009), using task-related functional MRI (Furl et al., 2011), and with voxel based morphometry to look at gray matter abnormalities (Garrido et al., 2009). Our aim was to conduct analyzes of white-matter integrity in these subjects to offer a comprehensive description of a large cohort of subjects with DP, and to investigate whether a deficit in local rather than long-range connections in the ventral temporal lobe was associated with developmental prosopagnosia.

## 2 Materials and methods

### 2.1 Participants

Sixteen individuals with DP and 16 age-matched controls volunteered for this study. We have previously reported analyzes of their behavioral data (Dalrymple et al., 2014b; Garrido et al., 2009), gray matter volume (Garrido et al., 2009), and functional responses (Furl et al., 2011). The current study includes the same participants listed in Garrido et al. (2009) except for one DP (DP14) and two controls (C4 and C6) whose DWI scans were suboptimal due to technical problems. For FFA fibers, we used for the tracking the face-specific functional regions of interest for these participants, which are reported in Furl et al. (2011). In particular, the right and left FFA were definable in 13 of the 16 DP participants and 15 of the 16 control participants.

The 16 DP participants (10 females) were between 20 and 46-years-old and had a mean age of 31 years (SD=8) while the 16 controls (10 females) had a mean age of 30 (SD=6). All participants were right-handed. All DP participants reported significant problems in recognizing faces in their daily lives, and each performed significantly below normal on two tests of face recognition: the Cambridge Face Memory Test (Duchaine and Nakayama, 2006) and a Famous Faces Test. Individual results on these tests and complete behavioral profiles are reported in Garrido et al. (2009).

Dimensionality reduction on behavioral performance measures was carried out using principal component analysis using Statistical Package for the Social Sciences 11.0 (SPSS Inc, Chicago, IL, USA) as described in Garrido et al. (2009). The four face identity recognition measures were the only measures to load highly on the first principle component, and therefore the participant loadings (factor scores) on this first component appear to provide a composite measure of facial recognition ability. Factor scores on the first component were found to be associated with gray matter density and face selectivity in the posterior fusiform gyrus and anterior temporal cortex (Garrido et al., 2009, Furl et al., 2011). Further, our factor scores capture variability in common with five facial identity recognition tasks while covarying out orthogonal sources of variability in three object recognition and three emotion recognition tasks. For these reasons, this first component was used as a measure of facial recognition ability in the current report. We have included a table in the supplementary section that lists individual scores on individual tests along with scores for this first component (Supplementary Table S1).

### 2.2 Scanning parameters

Scanning was conducted at the Wellcome Trust Center for Neuroimaging in London, UK. All MRI data were collected on a 3T Tim Trio scanner (Siemens Healthcare, Erlangen, Germany) using single-channel body coil excitation and a 12–channel receive-only head coil for acquisition. For diffusion data, a locally-implemented version (Nagy et al. 2007) of the twice-refocused spin echo diffusion sequence (Feinberg and Jakab, 1990, Reese et al., 2003) was collected twice. The two diffusion data sets were identical except the phase encoding blip direction was reversed to allow for adequate combination to correct susceptibility induced distortions (Andersson et al., 2003, Ruthotto et al., 2012) and vibration artifacts that were induced by fast switching of the large diffusion-encoding gradients (Gallichan et al., 2010, Mohammadi et al., 2012). Each diffusion data set contained images acquired using the following parameters: TE/TR=90/150 ms, FOV=220×220 mm^2^, 96×96 acquisition matrix, resolution=2.3×2.3×2.3 mm^3^, first 7 volumes at a *b*-value of 100 s/mm^2^ that were averaged to generate a low *b*-value volume followed by 61 brain volumes at a *b*-value of 1000 s/mm^2^ in 61 evenly-distributed directions. The protocol also included a 3D T1–weighted MDEFT image (Diechmann et al., 2004) (TE/TR=2.48/7.92 ms, FOV=256×240 mm^2^, 256×240 acquisition matrix, resolution=1x1×1 mm^3^).

### 2.3 Diffusion data analyzes

Prior to data analyzes, diffusion data were subject to state-of-the-art preprocessing methods to correct for artifacts common to echo-planar imaging acquisitions used in diffusion data. These include susceptibility-induced distortions, vibration artifacts, eddy current distortions, and participant motion. First, the two diffusion data sets with opposite phase-encoding blip directions that contain susceptibility-induced distortions in the opposite direction (Andersson et al., 2003) were corrected using a Hyperelastic Susceptibility Artifact Correction (HySCO) (Ruthotto et al., 2012), implemented in the open-source SPM toolbox ACID (Ruthotto et al. 2013) available at www.diffusiontools.com. The HySCO pre-processing routine here takes into account the need for the signal to be modulated by the Jacobi determinant of the deformation (Ruthotto et al., 2012, Ruthotto et al., 2013) and the COVIPER-method used here reduces the potential problem associated with redistributing signal as it uses the tensor-fit error to combine the data (Mohammadi et al., 2012). Signal drop-out that may result from vibration of the scanner couch (Gallichan et al., 2010) were corrected by an adequate combination (Mohammadi et al., 2012) of the two diffusion data sets with opposite phase-encoding blip directions. The resulting data set contained all 61 diffusion-weighted brain volumes and a low *b*-value brain volume. Next, in FSL (http://www.fmrib.ox.ac.uk), this dataset was corrected for residual eddy current distortions and participant motion. The diffusion-weighting vector directions (i.e. the b-vectors) were rotated as needed based on the motion correction parameters. Co-registration of the MDEFT high-resolution T1-weighted structural brain volume and the low *b*-value volume was performed in AFNI using the mutual information cost function (Cox, 1996). There were no significant differences between control and DP subjects in the SNR of low *b*-value brain volumes (*t*(*30*)=1.46, *p*>0.16)) nor in motion parameters for the DWI datasets (Euclidean norm) (*t*(*30*)=−1.18, *p*>0.24)).

#### 2.3.1 ILF and IFOF tractography: deterministic tractography

To isolate the ILF and IFOF, we used the same deterministic tractography parameters and guidelines followed by Thomas et al. (2009). User-defined ROIs were drawn by an investigator blinded to each participant's group. Tractography using these ROIs was performed by a separate investigator also blinded to each participant's group. As per Thomas et al. (2009), deterministic tractography was performed with a Fiber Assignment by Continuous Tracking (FACT) algorithm and a brute-force reconstruction approach, which uses all pixels in the entire brain volume as ‘seed’ pixels to generate the fibers. Fiber tracking was initiated by specifying three parameters: the minimum FA threshold for starting tracking (0.2), minimum FA for stopping tracking (0.2), and the curvature threshold (40°) for stopping tracking. A multiple ROI approach was used to define tracts in the following manner: A high-resolution T1-weighted brain volume was co-registered with the low *b*-value volume. The user-defined ROIs were defined on these images by one of the authors (A.S.) following the procedure outlined in Thomas et al. (2009). The tracts of interest were extracted and quantified in native space by another author (S.S.) using the protocol outlined in Thomas et al. (2009) to isolate the IFOF, ILF, forceps major (F-Ma), and forceps minor (F-Mi). As in Thomas et al. (2009), tracts generated from IFOF ROIs were removed from tracts generated by ILF ROIs, and tracts in the tapetum were removed from tracts generated from F-Ma ROIs. Like Thomas et al. (2009), the following metrics for the tracts of interest were calculated: percentage of fibers (% fibers), percentage of voxels (% voxels), and mean fractional anisotropy (mean FA) (Cook et al., 2006). We additionally analyzed mean diffusivity (MD), radial diffusivity (RD), and axial diffusivity (AD) because these metrics may be meaningful in describing microstructural differences in DP populations (Gomez et al., 2015).

As the parameters for deterministic tractography can affect tract reconstruction (Thomas et al., 2014) we recalculated percentage of fibers, percentage of voxels, and mean fractional anisotropy (mean FA) in tracts that had been defined using three additional curvature thresholds in the FACT-based algorithm (50°, 60°, 70°). Otherwise methods identical to those described above were employed.

#### 2.3.2 ILF and IFOF: deterministic and probabilistic tractography with group masks

In our cohort, we found that deterministic tracking methods led to non-specific tracts, and so we constructed group tract maps (Galantucci et al. 2011) and used these maps to mask out non-specific tracts. Group tract maps were thresholded to at least 50% of all participants to remove spurious tracts. These thresholds were based on visual inspection but were not specific to any one group as both groups were combined in this step. These group tract maps were returned to participant space and used to mask out non-specific tracts from the deterministic tract maps.

Probabilistic tractography may be better at tracking through crossing fibers than deterministic tractography so we also used probabalistic tractrography to assess the robustness of the deterministic tractography results. We recalculated percentage of voxels, and mean fractional anisotropy in tracts defined using probabilistic tractography (Bedpostx and Probtrackx from the FSL FDT toolbox, Behrens et al., 2003). We drew 5000 streamlines from each voxel in the ROI masks used above. Probabilistic tractography led to non-specific tracts, and so we constructed group probability maps for each tract (Galantucci et al., 2011) and used these group probability maps to mask out non-specific tracts. First, we thresholded individual probabilistic tract maps to at least 1000 streamlines, binarized these maps and warped them into standard space, and summed across individuals to create group probability maps. For ILF group maps, we first subtracted streamlines generated by the IFOF ROIs from streamlines generated by the ILF ROIs as was done for deterministic tractography. Group tract maps were thresholded to at least 50% of all participants to remove spurious tracts. These thresholds were not specific to any one group as both groups were combined in this step. These group tract maps were returned to participant space and used to mask out non-specific tracts from the probabilistic tract maps.

#### 2.3.3 FFA fibers: defined by face-specific functional regions of interest

Given the recent report that found differences in white matter (WM) properties within fibers defined by face-specific functional ROIs (Gomez et al., 2015), we used face-specific ROIs to define FFA fibers in our cohort. While Gomez et al. (2015) localized a putative sub-area of the FFA (the mFus/FFA-2), we used the peak coordinate of the FFA for tracking. Face-specific functional ROIs were based on data previously reported (Furl et al., 2011). The FFA peak was identified as the voxel in each individual with the maximum face-selectivity found within 10 mm of the peak face-selectivity observed at the group level (group level included the whole sample). Note, the tasks and scanning parameters used to define the functional ROIs here differ from those employed in Gomez et al. (2015). The FFA is conventionally observed as a unitary area that responds more to faces than non-face objects in localizer tasks. However, Weiner and colleagues have recently found that the FFA could be divided into sub-clusters of face selectivity, namely the ‘pFus’ or ‘FFA-1’ and the ‘mFus’ or ‘FFA-2’ (e.g., Weiner and Grill-Spector, 2012; Weiner et al., 2014). These sub-areas are observed using specialized surface coils. For our data, however, we did not observe the two clusters consistently and therefore used a more conventional definition of a unitary FFA.

As per Gomez et al. (2015), we extended spheres to WM to generate a seed region for tracking. We did so using an automated method that avoids potential bias in region placement. First, we drew a constant-sized sphere of 15 mm radius at the center coordinate of face-specific fROIs. We masked out areas of these spheres not located within the fusiform gyrus using an atlas-based mask registered to each subject's anatomical scan (Automated Anatomical Labeling (AAL) atlas; Tzourio-Mazoyer et al., 2002). We then determined the coordinates of the center of mass between overlap of this sphere and white matter with FA>0.2. We next drew a 10 mm sphere around these new coordinates and again determined the center of mass between overlap of this sphere and white matter with FA>0.2. Finally, we drew a 6 mm sphere around this center of mass and used this as the seed region for tractography. Tractography was conducted with probabilistic tractography using the AFNI FATCAT software (Taylor and Saad, 2013). Resultant tracts were thresholded to at least 10% of all drawn streamlines (1000 out of 10,000 per voxel). As in Gomez et al., 2015, we calculated whole bundle metrics (FA, MD, AD, RD) for FFA fibers as well as metrics for FFA fibers local to the fROIs. For local metrics, mean values were calculated for regions in FFA fibers that were within a 15 mm sphere drawn around the original seed region (Gomez et al., 2015). We also wanted to compare the spatial location of the local and whole bundle FFA fibers with those from the ILF and IFOF tracts. For consistency, we again defined ILF and IFOF with probabilistic tractography in AFNI FATCAT. Group tract maps were thresholded to at least 50% of all participants to remove spurious tracts. These thresholds were not specific to any one group as both groups were combined in this step. The spatial locations of the FFA fibers were compared to group masks of ILF and IFOF tract locations.

#### 2.3.4 ILF and IFOF tracts and FFA fibers: voxel-wise comparisons

We conducted voxel-wise comparisons of FA between groups within the tracts and fibers of interest. This overcomes limitations of tractography to distinguish tracts (Reveley et al., 2015) while minimizing the problem of multiple comparisons as compared to a whole brain search (whole brain voxel-wise comparisons in Supplementary Section 1). First, we made a mask that included ILF and IFOF tracts and FFA fibers by combining group masks of ILF and IFOF tracts and group masks of FFA fibers where at least 2 subjects had FFA fibers in the same location in standard space. This inclusive group threshold for FFA fibers was employed as peak voxels of functional ROIs used as starting points for tractography were in different locations in standard space and fibers would not necessarily align at a group level. This combined mask was dilated by one voxel to yield the final mask in which voxel-wise comparisons were conducted. Here we used the standard FA template in FSL as a group template (FSL TBSS, Smith et al., 2006). Note that data is resampled to voxels that are 1x1×1 mm^3^ in this step and hence, for voxel cluster extent thresholds, one voxel corresponds to 1 mm^3^ volume. In addition to FA, we compared MD, RD, and AD.

### 2.4 Statistical analyzes

For tractography dependent measures, either mixed design ANOVAs or independent *t*-tests were used to compare DP and control participants. For all *t*-values, accompanying two-tailed probabilities are reported in this manuscript. One-tailed probabilities are reported when significant with a-priori predictions based on findings from Thomas et al. (2009) or Gomez et al. (2015). Given the numerous analyzes necessary to verify prior findings, and that multiple measures of the same tract are highly correlated, we did not correct for the number of comparisons, as these are potentially overly conservative when measures are not independent, leading to Type II errors. Prior to the *t*-tests, homogeneity of variances was confirmed with Levene's test. For extended deterministic tractography, we added an additional factor of curvature threshold (40°, 50°, 60°, 70°) and compared groups using 2×2×4 mixed design ANOVAs with a between-participants factor of group (DP vs. control) and within-participants factors of brain hemisphere (Right vs. Left) and curvature threshold. Prior to ANOVAs, sphericity was confirmed using Mauchly's test. These statistical analyzes were performed in SPSS (SPSS Inc., Chicago IL).

For voxel-wise comparisons (FSL Randomise), we employed a liberal initial uncorrected threshold of *p*<0.005 followed by a cluster extent threshold of 40 voxels, as these thresholds have been shown in prior studies to be physiologically relevant (Boorman et al., 2007, Song et al., 2012). We additionally qualified our findings by calculating the corrected *p*-value for the cluster extents of identified regions by performing Monte-Carlo simulations to calculate the probability of finding a cluster of this size by random chance (AFNI AlphaSim; Cox, 1996). Monte-Carlo simulations with the smoothness (FWHM*x*=8.3 mm, FWHM*y*=11.7 mm, FWHM*z*=10.2 mm) and mask used demonstrated that of 10,000 random simulations, 500 random simulations at *p*<0.005 uncorrected contained significant clusters of at least 587 voxels. Hence the cluster extent threshold for a corrected *p*<0.05 is 587 voxels.

## 3 Results

### 3.1 ILF and IFOF tractography: deterministic tractography

**Fig. 1:**
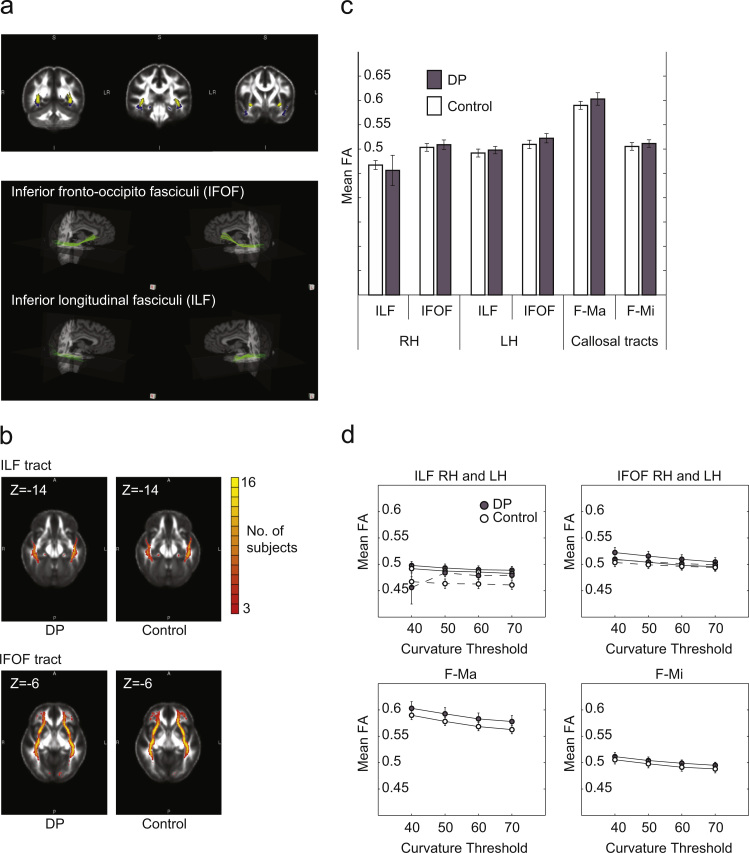
ILF and IFOF: Deterministic tractography. (a) At a group level (top), the relative trajectories of the ILF and IFOF through the temporal cortices shown here are visually similar to those depicted in Thomas et al. (2009). Streamlines generated for each individual were also checked visually (bottom). Here, the trajectories of the ILF and IFOF are shown here on top of the aligned anatomical volume in a single representative subject. These respective trajectories were visually similar to those for the ILF and IFOF as depicted in a diffusion tensor atlas (Catani and Thiebaut de Schotten, 2008). (b) ILF and IFOF tract maps were transformed into standard space and overlaid to generate group maps of tract trajectories for the DP and control groups. The numbers of participants with at least one streamline passing through the voxel is indicated by color scale according to legend. (c) The mean FA in ILF and IFOF tracts as well as control callosal tracts showed no statistically significant differences between the two groups for any of the tracts tested (Table 1). Note that the mean FA values for controls (mean age=30) in this report are comparable to those reported for younger control subjects depicted in Thomas et al. (2008) and only slightly greater than values reported for mean FA values for older control subjects (mean age=56) depicted in Thomas et al. (2009) as would be expected given known age-related decline (Thomas et al., 2008). (d) We additionally performed deterministic tractography at various curvature thresholds to test the robustness of our finding across different methods of tract identification. Plotted here are mean FA values for ILF and IFOF tracts isolated at various curvature thresholds with right hemisphere values connected by dotted lines, and left hemisphere values connected by solid lines. Again, no significant group differences were found for any of the metrics (Table 3). Similarly, for control callosal tracts in the F-Ma and F-Mi, no significant group differences were found for any of the metrics (Table 3).

Using the deterministic tractography methods described in Thomas et al. (2009), the relative trajectories of the ILF and IFOF in ventral temporal cortex were visually comparable to the trajectories shown by Thomas et al. (2009) and Catani and Thiebaut de Schotten (2008) (Fig. 1a). As in Thomas et al. (2009), the majority of control participants had prominent and visible tracts in the ILF and IFOF (Fig. 1b right). However, the majority of DP participants also had prominent and visible tracts in the ILF and IFOF (Fig. 1b left). Comparisons of mean fractional anisotropy (FA) revealed no significant differences between participants with DP and controls in any of the tracts tested including right ILF, right IFOF, left ILF, left IFOF or in the control callosal tracts F-Ma and F-Mi (Fig. 1c, Table 1). Neither did we find any significant correlations between mean FA in any of the tracts with face recognition ability (Table 2). Hence, for FA measures, we did not replicate Thomas et al. (2009) and could not reject the null hypothesis when testing for group differences. Inter-individual variability in DP subjects for FA is plotted in Supplementary Fig. S1.

In addition to FA, we also looked at measures of density and volume of fibers as in Thomas et al. (2009). We again could not replicate the previous findings and found no statistically significant group differences for any of the tracts of interest for % fibers and % volume (Table 1). Neither was there a correlation between any of these measures and face recognition ability (Table 2). Inter-individual variability in DP subjects for these measures is plotted in Supplementary Fig. S1. Finally, no statistically significant group differences for any of the tracts of interest were found for MD, AD, and RD measures (Supplementary Table S2).

As deterministic tractography is sensitive to curvature thresholds set prior to tracking (Thomas et al., 2014), we also employed three additional curvature thresholds for tracking (50°, 60°, 70°) along with the 40° employed by Thomas et al. (2009). Again, no group differences were found. A 2×4×2 (Group by Curvature by Hemisphere) mixed design ANOVAs did not show a significant main effect of Group for ILF and IFOF tracts for mean FA, % fibers or % volume (Fig. 1d, Table 3). Additionally, 2×4 (Group by Curvature) mixed design ANOVAs showed no significant main effects of group for control callosal tracts (Fig. 1d, Table 3).

### 3.2 ILF and IFOF: deterministic and probabilistic tractography with group masks

**Fig. 2:**
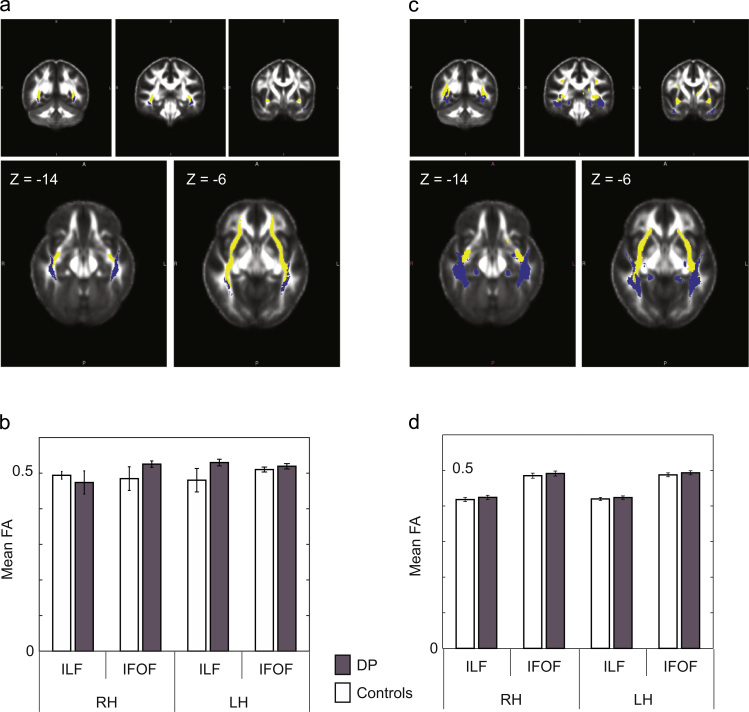
ILF and IFOF: deterministic and probabilistic tractography with group masks. Both deterministic and probabilistic tractography resulted in non-specific tracts, and so we constructed group probability maps for each tract (Galantucci et al., 2011) and used these group probability maps (at least 50% of subjects) to mask out non-specific tracts. (a) The relative trajectories of this mask of ILF and IFOF tracts for deterministic tractography. (b) Fractional anisotropy in ILF and IFOF tracts showed no statistically significant differences between the two groups for any of the tracts (Table 4). (c) The relative trajectories of this mask of ILF and IFOF tracts for probabilistic tractography. (d) Again, no significant group differences were found in fractional anisotropy for any of the tracts (Table 4).

Both deterministic and probabilistic tractography resulted in non-specific tracts, and so we constructed group tract maps (Galantucci et al., 2011) and used these group maps to mask out non-specific tracts. The relative trajectories of these masks of ILF and IFOF tracts with both deterministic (Fig. 2a) and probabilistic tractography (Fig. 2b) were visually similar to the trajectories depicted in a diffusion tensor atlas (Catani and Thiebaut de Schotten, 2008). We again failed to reveal significant group differences in mean FA for right ILF, right IFOF, left ILF and left IFOF with both deterministic and probabilistic tractography (Fig. 2b and d, Table 4) and failed to show significant correlations with face recognition ability for right ILF, right IFOF, left ILF and left IFOF (Table 5). Inter-individual variability in DP subjects for FA is plotted in Supplementary Fig. S2. The same was true for % volume (Table 4, Table 5).

### 3.3 FFA fibers: defined by face-specific functional regions of interest

**Fig. 3:**
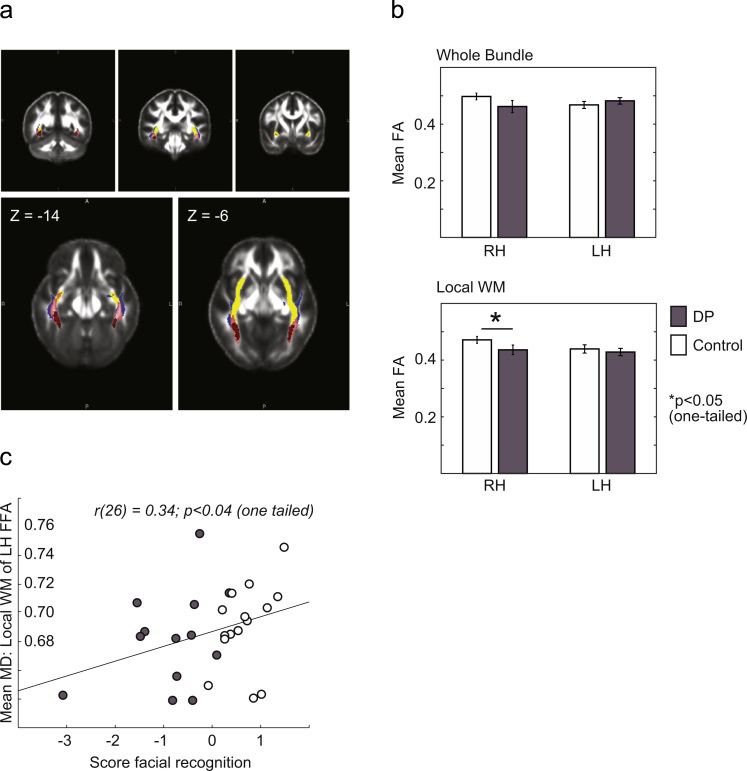
FFA fibers: Defined by face-specific functional regions of interest. (a) On the group level, WM regions of FFA fibers local to fROIs (local WM, in red; color range dark to light=3 to 28 subjects) were centered on posterior sections of the whole bundle of FFA fibers (in blue; color range dark to light=3 to 28 subjects). In posterior regions of the brain (left), FFA fibers were more ventral to ILF (in blue) and IFOF fibers (in yellow) although there was partial overlap. Moving more anterior (middle to right), FFA fibers began to show increasing spatial overlap with ILF fibers. (b) Compared to control subjects, subjects with DP demonstrated lower mean FA (local WM) in right FFA fibers (Table 6). (c) A significant correlation between MD (local WM) in left FFA fibers and face recognition ability was found across both DP and control subjects (Supplementary Table S4). (For interpretation of the references to color in this figure legend, the reader is referred to the web version of this article.)

On the group level, WM regions of FFA fibers local to fROIs (local WM, Fig. 3a in red) were centered on the posterior section of the whole bundle of FFA fibers (Fig. 3a in blue). The FFA fibers partially overlapped with ILF tracts but were more ventrally located in posterior regions of the brain and became more spatially overlapping in anterior regions of the brain (Fig. 3a). This is comparable to the description of FFA fibers in Gomez et al. (2015). For whole bundle FFA fibers, no group differences were found for FA (Table 6), nor were found any correlations with behavior (Table 7). For local WM FFA fibers, we found lower FA values in DP compared to controls in the right FFA (*p*<0.05, one-tailed, Table 6). There were no correlations with behavior (Table 7). Inter-individual variability in DP subjects for FA is plotted in Supplementary Fig. S3.

There were no group differences for MD, AD and RD measures (Supplementary Table S3) although there was a significant positive correlation between MD in the left FFA and face recognition ability across control and DP subjects (*p*<0.04, one-tailed) (Fig. 3c, Supplementary Table S4). Within group correlations were not significant.

### 3.4 ILF and IFOF tracts and FFA fibers: voxel-wise comparisons

**Fig. 4:**
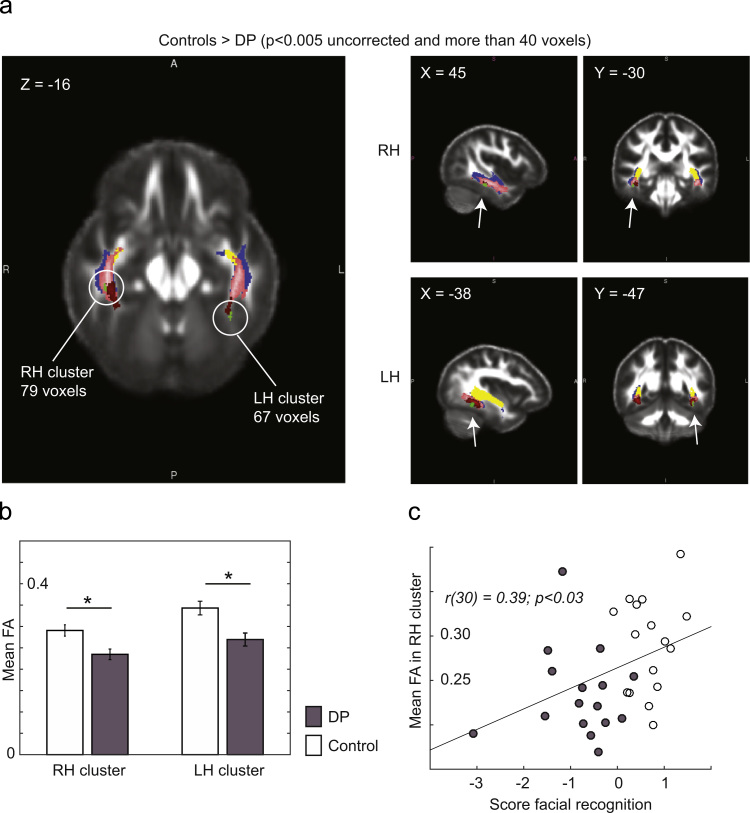
: ILF and IFOF tracts and FFA fibers: voxel-wise comparisons. (a) For FA measures, voxel-wise comparisons demonstrated that at *p*<0.005 uncorrected followed by a cluster extent threshold of 40 voxels, two regions emerged past this threshold for FA measures with Controls>DP (in green). Also shown in this figure are local WM FFA fibers (in red), whole bundle FFA fibers (in pink), ILF tracts (in blue) and IFOF tracts (in yellow). (b) As expected given that these regions were extracted based on significant differences, FA within these clusters was greater in control compared to DP subjects. (c) A significant correlation was found between FA measures in the RH region and face recognition ability across control and DP subjects (*p*<0.03). (For interpretation of the references to color in this figure legend, the reader is referred to the web version of this article.)

We conducted voxel-wise comparisons of FA between groups within the tracts and fibers of interest with the mask including the ILF and IFOF tracts and FFA fibers. This mask was dilated by one voxel to account for imperfect alignment. At a threshold of *p*<0.005 uncorrected followed by a cluster extent threshold of 40 voxels (Boorman et al., 2007, Song et al., 2012), two regions emerged past this threshold for FA measures with Controls>DP (in green in Fig. 4a). Importantly, these two regions were overlapping with or adjacent to local WM regions of the FFA (in red in Fig. 4a). FA measures within these clusters were extracted for all subjects with expected differences in FA between Control and DP subjects for both the RH (*t*(30)=3.01, *p*<0.005) and LH (*t*(30)=3.33, *p*<0.002) regions (Fig. 4b). Inter-individual variability in DP subjects for FA is plotted in Supplementary Fig. S4. To qualify these findings, we used Monte-Carlo simulations with the smoothness (FWHM*x*=8.3 mm, FWHM*y*=11.7 mm, FWHM*z*=10.2 mm) and mask used to calculate the probability of finding a cluster of this size by random chance. For the RH cluster, a cluster of 79 voxels was found in 59.8% of 10,000 random simulations at an uncorrected *p*<0.005, for a corrected *p*=0.60. For the LH cluster, a cluster of 67 voxels was found in 63.9% of 10,000 random simulations at an uncorrected *p*<0.005, for a corrected *p*=0.64.

A significant correlation was found between FA measures in the RH region and face recognition ability across control and DP subjects (*p*<0.03) (Fig. 4c). This correlation was not significant for the LH region (*p*=0.22).

For DP>Control in FA measures, one RH cluster emerged that was near the posterior end of the bundle of FFA fibers (Supplementary Fig. 4f). Additionally, clusters emerged for comparisons for MD, AD, and RD. Notably, differences were found for MD and RD in regions overlapping with right local WM FFA fibers (Supplementary Fig. 4c–e).

We also conducted voxel-wise comparisons across the whole brain. This is discussed in Supplementary Section 1.

## 4 Discussion

Prior studies using diffusion tensor imaging in small samples of subjects with DP (*n*=6 or *n*=8) offer conflicting views on the neurobiological bases for DP. Here, we addressed these inconsistent findings in a sample of subjects with DP that is larger than both prior studies combined (*n*=16) using a comprehensive set of analyzes that included tractography-based measures for long-range tracts and functionally defined FFA fibers, as well as voxel-based comparisons within tracts and fibers of interest. We found no statistically significant differences on any measure of white matter integrity between the two groups for both the ILF and the IFOF and no relationships with behavior (Fig. 1, Fig. 2, Table 1, Table 2, Table 3, Table 4, Table 5). We found evidence to support an alternative hypothesis focused on fibers local to face-specific fROIs in the fusiform gyrus similar to those found by Gomez et al. (2015). Specifically, DP subjects had lower FA in WM local to the right FFA (Fig. 3, Table 6, Table 7). Moreover, using voxel-wise comparisons within tracts and fibers of interest, two regions that showed greater FA in controls compared to DPs were co-localized with local WM regions in FFA fibers bilaterally (Fig. 4). This finding is important given recent studies highlighting inherent limitations of DTI to distinguish tracts and fibers with tractography alone (Thomas et al., 2014, Reveley et al., 2015). Further, we found correlations between FA measures in right FFA fibers and face recognition ability and between MD measures in left FFA fibers with face recognition ability (Figs. 3c and 4c). Note that our null and our positive results applied the same statistical criterion. As we conducted several more comparisons on the ILF and IFOF fibers than on the FFA fibers, and yet found differences only in the latter, it is unlikely that this dissociation is simply the result of Type I errors stemming from multiple comparisons.

While our results are broadly similar to those from Gomez et al. (2015), they differ from the previous report in notable ways. Gomez et al. (2015) did not find lower FA values in their DP subjects compared to controls and instead found lower MD values in local WM bilaterally and in the whole bundle for right FFA fibers. In contrast, we found evidence that subjects with DP had lower FA in or near local WM bilaterally (Fig. 3, Fig. 4) and not in the whole bundle, as well as identifying MD and RD differences in right local WM (Supplementary Fig. S4c and d). Gomez et al. (2015) found FA in local WM within right FFA positively correlated with face recognition ability in healthy controls. Here, we found positive correlations between MD in local WM within left FFA and face recognition ability (Fig. 3c) and between FA in local WM within right FFA and face recognition ability across DP and controls when both groups were collapsed together (Fig. 4c). Our ability to find FA differences and correlations that Gomez et al. (2015) did not may be due to the fact that our study had more subjects and hence more statistical power, or due to the addition of voxel-wise comparisons within tracts and fibers of interest that could localize regions of greatest difference between groups (Fig. 4). Another possibility is that we used a different task and method for functionally defining our ROIs (Furl et al., 2011). Further, due to the complexity of neuroimaging, it is very unlikely for any two given neuroimaging studies to perfectly replicate (Fletcher and Grafton, 2013). Finally, DP is a heterogeneous disorder (Susilo and Duchaine, 2013). Irrespective of these differences or perhaps notable because of them, the findings of these two reports using different cohorts, different scanning parameters, different functional tasks to localize functional ROIs, and differing behavioral methods have some striking similarities along the following lines: FA and MD values in local WM in FFA fibers show group differences and correlations with face recognition ability.

These conclusions are contrary to those of Thomas et al., (2009), who found differences in long-range tracts, notably ILF and IFOF bilaterally. What might account for these differences? One difference is that our sample was younger than their sample (mean age of 31 *versus* 58). Statistical inference is based on the concept that random sampling from a population can be used to infer properties about the population. In addition to a large sample size, scientific studies typically aim to reduce sources of heterogeneity when making causal inference as heterogeneity limits the ability to make valid inference (Xie, 2013). Normal, healthy aging is known to increase both cognitive heterogeneity (Ardila 2007) and increase heterogeneity in white matter integrity due to heterogeneous age-related breakdown of white matter (Bartzokis et al., 2004) including heterogeneous age-related breakdown of microstructural integrity of the IFOF (Thomas et al., 2008). In contrast, our (mean age of 31) and Gomez's (mean age of 34) studies had a younger sample of DP participants. Another possibility is that DP subjects have greater age-related decline in ILF and IFOF than normal subjects.

Methodological and imaging issues such as variations in tractography methods or in eddy currents, vibration artifacts, or susceptibility distortions may also explain differences in findings. As compared to Thomas et al. (2009) we used more updated scanning protocols along with more extensive tractography analyzes. Recent papers by Thomas et al. (2014) and Reveley et al. (2015) demonstrated the inherent limitations of any tractography method in sensitivity and accuracy. Coupled with the limited scanning parameters (6 directions), the deterministic tractography method used by Thomas et al. (2009) was low on sensitivity for detecting real tracts (Thomas et al., 2014), and it is possible that this sensitivity issue was more pronounced in some tracts (such as IFOF and ILF) versus others (such as Forceps Major and Minor) given differing relationships in sensitivity between tracts and tracting algorithms (Thomas et al., 2014). For this reason, we used several tractography methods including deterministic with various curvature thresholds, and probabilistic tractography both with and without group masks, and we found the same lack of group differences across all analyzes for the IFOF and ILF.

The DTI results from Thomas et al. (2009) have been used to support a general hypothesis that DP is best conceptualized as a posterior–anterior disconnection syndrome (Behrmann and Plaut, 2013). According to this hypothesis, individuals with DP have intact face processing in posterior occipito-temporal areas, as evidenced by normal face-selectivity and repetition suppression in these regions (Avidan et al., 2005, Avidan et al., 2011; Hasson et al., 2003), but have face recognition deficits due to poor communication between these posterior areas and the anterior temporal cortex due to reduced integrity in the ILF and IFOF tracts (Thomas et al., 2009). Our current findings showing intact ILF and IFOF integrity in DP are inconsistent with this posterior–anterior disconnection account. Further, a number of previous studies indicate that posterior occipito-temporal areas are not functioning normally in many people with DP. While face-selective regions in occipito-temporal cortex are present in most participants with DP (e.g. Avidan et al., 2005), we found that these posterior regions show reduced face selectivity in DPs as compared to controls (Furl et al., 2011; but see Avidan et al., 2014). Some DP participants produce early event-related electromagnetic responses at occipito-temporal sensors with reduced face-selectivity (Bentin et al., 1999, Harris et al., 2005, Towler et al., 2012) and, one study found that, unlike controls, a majority of participants with DP do not show a stronger response at these sensors to inverted compared to upright faces (Towler et al., 2012). Complementing these findings, structural analyzes have found gray-matter abnormalities in posterior temporal cortex (Garrido et al., 2009; but see Behrmann et al., 2007). The current report further suggests that white matter microstructural abnormalities in the ventral temporal cortex are mainly found in regions local to where functional and gray-matter abnormalities in posterior temporal cortex have been previously described (Furl et al., 2011, Garrido et al., 2009). These results suggest that dysfunction in posterior regions is often present in DP.

Studies in healthy controls have found links between facial recognition ability and FA in the ILF (Postans et al., 2014) or with FA in anterior but not posterior portions of the ILF (Tavor et al., 2014), which on first glance is contrary to the findings of Gomez et al., 2015 and the current report. One explanation for this discrepancy was discussed by Gomez et al. (2015), who pointed out that fibers local to the FFA, while distinct from ILF fibers and localized more ventrally in posterior sections of the tract, become increasingly spatially overlapping with the ILF in more anterior portions of the brain. We also found this pattern in the current report (Fig. 3, Fig. 4). In other words, FFA fibers and ILF fibers are difficult to differentiate particularly in anterior regions. Another interpretation based on autoradiographic studies in non-human primates is that the ILF is not in fact a long-range tract, but rather a series of U fibers connecting adjacent regions in occipito-temporal regions (Tusa and Ungerleider, 1985). In other words, the ILF may be a collection of short-range fibers including FFA fibers and many other fibers that collectively form the tract. Unfortunately, diffusion weighted imaging based tractography is inherently limited in its ability to conclusively differentiate between short-range fibers and long-range tracts (Reveley et al., 2015, Thomas et al., 2014). In other words, these interpretations cannot be well differentiated with current tractography methods in diffusion-weighted imaging. For this reason, we added an analysis that did not rely on the ability of tractography to differentiate tracts, and instead made a mask of regions that belonged to either the FFA fibers or long-range ILF and IFOF tracts and conducted voxel-wise comparisons within this mask. We again found differences bilaterally that co-localized with local WM to FFA fibers. This latter finding suggests that differences between groups are in fibers local to functionally defined face-specific regions irrespective of tractography limitations. This method of initial tractography followed by voxel-wise comparisons within tracts and fibers of interest may be one method of offering convergent evidence to overcome some of the limitations inherent in tractography.

The current report is the first to look at all three fiber/tract types implicated in DP (FFA fibers, ILF, and IFOF tracts) and also included more subjects with DP (*n*=16) than both prior studies combined ( *n*=6 in Thomas et al., 2009; *n*=8 in Gomez et al., 2015). Along with other reports detailing behavior (Dalrymple et al., 2014b, Garrido et al., 2009), task-related functional responses (Furl et al., 2011), and gray matter volume (Garrido et al. 2009), the analyzes of white-matter integrity in these subjects described here offers a comprehensive view of a large cohort of subjects with DP. Our results suggest group differences and correlations with face recognition ability in local WM in posterior regions of FFA fibers near the face-specific regions of the fusiform gyrus and not along the whole bundle that contained anterior regions of FFA fibers, and not in any of the ILF and IFOF tracts. Given posterior regions with reduced face selectivity in these DPs as compared to controls (Furl et al., 2011), gray-matter abnormalities in posterior temporal cortex (Garrido et al., 2009), and the current findings, all of which correlated with behavioral measures of poor face recognition, deficits local to posterior regions rather than disconnection along major tracts may more likely relate to developmental prosopagnosia. In contrast, non face-specific impairments in a wide variety of disorders including psychosis (Hatton et al., 2014), Alzheimer's disease (Meng et al., 2012, Kitamura et al., 2013), and language deficits (Dick et al., 2013) has been linked to WM integrity in ILF and IFOF tracts, suggesting these tracts may play a wide role in cognition. Patient cases where ILF deficits are found in addition to face-processing deficits are also often accompanied by extensive atrophy in gray matter making it difficult to differentiate between the role of white and gray matter (Grossi et al., 2012).

This point highlights that subtle differences may only be resolvable with targeted methods such as using functional ROIs for tractography followed by voxel-wise comparisons in tracts and fibers of interest. Tractography is limited in its ability to accurately define tracts with specificity and/or sensitivity (Thomas et al., 2014, Reveley et al., 2015), while voxel-wise comparisons are limited in their ability to detect small local differences that can overcome correction for family-wise error even when the search is within a targeted mask (Fig. 4) and at a whole brain level may be insufficient to differentiate between Type 1 and 2 errors (Supplemental Section One). The combination of both methods, along with targeted comprehensive analyzes aimed at verifying prior claims using larger cohorts as performed here may be necessary to converge upon the true nature of structural brain abnormalities associated with a behavioral deficit. Given the importance of drawing reliable conclusions from clinical neuroimaging and at the same time, the limitations inherent to neuroimaging methods (Thomas et al., 2014, Reveley et al., 2015), convergent evidence using several methods within a single report, and verification of findings across studies in large cohorts may be the optimal way of employing imaging to inform understanding of a disorder (Fletcher and Grafton, 2013).

## Figures and Tables

**Table 1:** ILF and IFOF: deterministic tractography; independent *t*-tests comparing DP and control groups.

Measure	Tract	*t*-value^a^ (dof=30)	*p*-value
Fractional anisotropy (Fig. 1c; Fig. 2e,f)	Right ILF	0.34	0.74
Right IFOF	−0.43	0.67
Left ILF	−0.54	0.59
Left IFOF	−0.99	0.33
Forceps major	−0.86	0.40
Forceps minor	−0.54	0.59
% Fibers (Fig. 2a,b)	Right ILF	−0.25	0.80
Right IFOF	1.50	0.15
Left ILF	0.22	0.83
Left IFOF	0.77	0.45
F-Ma	0.33	0.74
F-Mi	0.96	0.34
% Volume (Fig. 2c,d)	Right ILF	−0.23	0.82
Right IFOF	1.54	0.13
Left ILF	0.07	0.95
Left IFOF	0.54	0.60
F-Ma	0.05	0.86
F-Mi	0.46	0.65

a Positive values indicate control>DP while negative values indicate DP>control

**Table 2:** ILF and IFOF: deterministic tractography; correlation with face recognition ability.

Measure	Tract	*r*-value (dof=30)	*p*-value
Fractional anisotropy	Right ILF	−0.01	0.98
Right IFOF	0.03	0.87
Left ILF	0.01	0.97
Left IFOF	−0.10	0.58
% Fibers	Right ILF	−0.09	0.63
Right IFOF	0.05	0.80
Left ILF	−0.10	0.60
Left IFOF	0.24	0.19
% Volume	Right ILF	−0.09	0.63
Right IFOF	0.08	0.68
Left ILF	−0.12	0.53
Left IFOF	0.16	0.37

**Table 3:** ILF and IFOF: deterministic tractography with various tracking curvature thresholds; mixed-design ANOVAs (main effect of group).

Measure	Tract	*F*-value (*F*(*1*,*30*))	*p*-value
Fractional anisotropy	ILF	0.69	0.41
IFOF	0.52	0.48
(Fig. 3)
F-Ma	1.04	0.32
F-Mi	0.43	0.52
% Fibers	ILF	0.01	0.93
IFOF	1.55	0.22
F-Ma	0.65	0.43
F-Mi	1.65	0.21
% Volume	ILF	0.001	0.98
IFOF	0.88	0.36
F-Ma	0.40	0.53
F-Mi	0.24	0.63

**Table 4:** ILF and IFOF: deterministic and probabilistic tractography with group masks; independent *t*-tests comparing DP and control groups.

Measure	Tract	*t*-value^a^ (dof=30)	*p*-value
Deterministic:	Right ILF	0.59	0.56
Fractional anisotropy
Right IFOF	−1.18	0.25
(Fig. 2c)
Left ILF	−1.44	0.16
Left IFOF	−0.90	0.38
Deterministic:	Right ILF	−0.34	0.82
Right IFOF	0.70	0.49
% Volume
Left ILF	0.23	0.82
Left IFOF	0.88	0.38
Probabilistic:	Right ILF	−0.74	0.47
Fractional anisotropy
Right IFOF	−0.61	0.54
(Fig. 2d)
Left ILF	−0.60	0.55
Left IFOF	−0.72	0.48
Probabilistic:	Right ILF	0.11	0.92
% Volume	Right IFOF	0.36	0.72
	Left ILF	−0.27	0.79
Left IFOF	1.11	0.27

a Positive values indicate control>DP while negative values indicate DP>control

**Table 5:** ILF and IFOF: deterministic and probabilistic tractography with group masks; correlation with face recognition ability.

Measure	Tract	*r*-value (dof=30)	*p*-value
Deterministic:	Right ILF	0.02	0.94
Right IFOF	−0.18	0.32
Fractional anisotropy
Left ILF	−0.15	0.41
Left IFOF	−0.07	0.70
Deterministic:	Right ILF	−0.19	0.29
Right IFOF	−0.07	0.72
% Volume
Left ILF	−0.114	0.54
Left IFOF	0.08	0.66
Probabilistic:	Right ILF	0.01	0.96
Right IFOF	0.01	0.95
Fractional anisotropy
Left ILF	−0.02	0.93
Left IFOF	−0.03	0.89
Probabilistic:	Right ILF	−0.07	0.70
% Volume
Right IFOF	0.05	0.77
Left ILF	−0.18	0.32
Left IFOF	0.20	0.26

**Table 6:** FFA fibers: defined by face-specific functional regions of interest; independent *t*-tests comparing DP and control groups.

Measure	Tract	*t*-value^a^ (dof=26)	*p*-value
Whole bundle: Fractional anisotropy	Right FFA	1.51	0.14

Left FFA	−0.84	0.41
Local WM: Fractional anisotropy	Right FFA	1.73	0.096; *<0.05*
^⁎^one tailed
(Fig. 3b)
Left FFA	0.56

0.58

a Positive values indicate control>DP while negative values indicate DP>control

**Table 7:** FFA fibers: Defined by face-specific functional regions of interest**;** Correlation with face recognition ability.

Measure	Tract	*r*-value (dof=26)	*p*-value
Whole bundle: Fractional anisotropy	Right FFA	0.21	0.29
Left FFA	−0.36	0.86
Local WM: Fractional anisotropy	Right FFA	0.22	0.25
	Left FFA	0.09	0.64
