# Supplementary material for: Local but not long-range microstructural differences of the ventral temporal cortex in developmental prosopagnosia
Source: Neuropsychologia. 2015 Nov;78:195–206. doi: 10.1016/j.neuropsychologia.2015.10.010 (PMC4640146; doi:10.1016/j.neuropsychologia.2015.10.010)
Supplement: Supplementary file 1 — Supplementary material [file mmc1.doc]

**Supplementary Table S1: Relationships between behavioral metrics in all participants**

| **Participant** | **CFMT** | **Baldwomen** | **Famous Faces Ratio** | **PCA Factor 1** |
| --- | --- | --- | --- | --- |
| **C1** | 68 | 1.00 | 0.86 | 1.35194 |
| **C2** | 66 | 0.94 | 0.95 | 1.48366 |
| **C3** | 69 | 1.00 | 0.85 | 0.85373 |
| **C5** | 69 | 1.00 | 0.98 | 1.13633 |
| **C7** | 58 | 0.99 | 0.93 | 0.25858 |
| **C8** | 72 | 0.99 | 0.90 | 1.01604 |
| **C9** | 63 | 0.99 | 0.89 | 0.67442 |
| **C10** | 60 | 0.97 | 0.77 | -0.07963 |
| **C11** | 61 | 0.99 | 0.95 | 0.76096 |
| **C12** | 60 | 1.00 | 0.88 | 0.53495 |
| **C13** | 63 | 0.97 | 0.80 | 0.76442 |
| **C14** | 59 | 0.99 | 0.69 | 0.40704 |
| **C15** | 70 | 0.96 | 0.91 | 0.25977 |
| **C16** | 55 | 0.99 | 0.59 | 0.37966 |
| **C17** | 69 | 0.98 | 0.91 | 0.72514 |
| **C18** | 60 | 0.96 | 0.97 | 0.21155 |
| **DP1** | 36 | 0.86 | 0.33 | 0.3539 |
| **DP2** | 43 | 0.86 | 0.49 | 0.09442 |
| **DP3** | 35 | 0.93 | 0.38 | -0.36704 |
| **DP4** | 37 | 0.89 | 0.46 | -0.56969 |
| **DP5** | 32 | 0.69 | 0.25 | -1.48124 |
| **DP6** | 40 | 0.88 | 0.35 | -0.72793 |
| **DP7** | 37 | 0.88 | 0.44 | -0.40356 |
| **DP8** | 32 | 0.93 | 0.58 | -0.42888 |
| **DP9** | 37 | 0.83 | 0.58 | -0.81651 |
| **DP10** | 26 | 0.52 | 0.04 | -3.07769 |
| **DP11** | 34 | 0.75 | 0.02 | -0.31183 |
| **DP12** | 36 | 0.77 | 0.34 | -1.54699 |
| **DP13** | 28 | 0.79 | 0.47 | -1.38974 |

**Supplementary Table S2:**

ILF and IFOF: Additional results of deterministic tractography: Independent t-tests comparing DP and control groups

| Measure | Tract  (Control Mean/SD; DP Mean/SD | t-value^a^ (dof=30) | p-value |
| --- | --- | --- | --- |
| Axial  Diffusivity (AD) | right ILF (1.07/0.05;1.01,0.27)  right IFOF (1.11/0.04; 1.12,0.05)  left ILF (1.12,0.05; 1.14,0.05)  left IFOF (1.15/0.05; 1.16/0.04) | 0.77  -1.14  -0.69  -0.85 | 0.45  0.26  0.50  0.40 |
| Radial  Diffusivity (RD) | right ILF (0.49/0.24;0.45/0.12)  right IFOF (0.47/0.23; 0.47/0.29)  left ILF (0.49/0.03; 0.49/0.03)  left IFOF (0.48/0.23; 0.47/0.03) | 1.3  0.03  -0.02  0.57 | 0.20  0.98  0.98  0.57 |
| Mean  Diffusivity (MD) | right ILF (0.68/0.23; 0.63/0.17*10^3)  right IFOF (0.67/0.20; 0.68/0.18^10^3)  left ILF (0.70/0.21; 0.70/0.22*10^3)  left IFOF (0.69/0.17; 0.69/0.18*10^3) | 0.99  -0.77  -0.47  -0.07 | 0.33  0.45  0.64  0.94 |

^a^ positive values indicate control > DP while negative values indicate DP > control

**Supplementary Table S3:**

FFA fibers: Additional results; Independent t-tests comparing DP and control groups

| Measure | Tract (Control Mean/SD; DP Mean/SD) | t-value^a^  (dof = 26) | p-value |
| --- | --- | --- | --- |
| Whole bundle: MD | right FFA (0.66/0.03; 0.67/0.03*10^3)  left FFA (0.68/0.03;0.68/0.03*10^3) | -0.639  0.179 | 0.53  0.86 |
| Whole Bundle: AD | right FFA (1.04/0.04;1.05/0.09)  left FFA (1.07/0.09; 1.08/0.07) | -0.535  -0.277 | 0.60  0.78 |
| Whole Bundle: RD | right FFA (0.47/0.03; 0.48/0.03)  left FFA (0.48/0.03; 0.48/0.03) | -1.550  0.637 | 0.13  0.53 |
| Local WM:  MD | right FFA (0.66/0.02; 0.67/0.04*10^3)  left FFA 0.69/0.03;0.68/0.03*10^3) | -1.0  0.73 | 0.32  0.47 |
| Local WM: AD | right FFA(1.04/0.07; 1.01/0.06)  left FFA (1.05/0.88;1.03/0.06) | 0.96  0.89 | 0.35  0.38 |
| Local WM: RD | right FFA (0.46/0.03;0.48/0.07)  left FFA (0.51/0.03; 0.51;0.04) | -1.49  0.01 | 0.15  > 0.99 |

^a^ positive values indicate control > DP while negative values indicate DP > control

**Supplementary Table S4: FFA fibers:** Correlation with face recognition ability

| Measure | Tract | r-value (dof = 26) | p-value |
| --- | --- | --- | --- |
| Whole bundle: MD | right FFA  left FFA | 0.14  0.34 | 0.48  0.24 |
| Whole bundle: AD | right FFA  left FFA | -0.10  0.14 | 0.61  0.48 |
| Whole Bundle: RD | right FFA  left FFA | -0.06  0.17 | 0.76  0.38 |
| Local WM: MD  (Figure 3c) | right FFA  left FFA | 0.09  0.34 | 0.67  0.08; *<0.04 one-tailed** |
| Local WM: AD | right FFA  left FFA | 0.24  -0.28 | 0.21  0.15 |
| Local WM: RD | right FFA  left FFA | -0.10  0.15 | 0.62  0.46 |

**Supplementary Section 1: Whole-brain voxel-wise comparisons**

Here, we highlight that for small differences in local WM, a whole brain search may not be appropriate due to a large increase in family-wise error. We conducted whole-brain voxel-wise comparisons within a skeletonized mask (threshold mean FA > 0.2) for FA (FSL TBSS, Smith *et al*., 2006), mean anisotropy in partial volumes anisotropy f1 and f2 from BedpostX (FSL TBSSx, Jbabdi *et al.*, 2010), as well as for MD, AD, and RD. For the skeletonized mask, Monte-Carlo simulations with the smoothness (FWHMx = 8.3 mm, FWHMy = 11.7 mm, FWHMz = 10.2 mm) and whole brain skeletonized mask used demonstrated that at an initial threshold of p<0.005 uncorrected, a cluster extent threshold necessary for a corrected p<0.05 was 259 voxels. No voxels anywhere in the brain reached this corrected threshold. The largest cluster to emerge from any comparison within the mask was a cluster of 115 voxels which had a corrected p = 0.54 and was for AD in the superior longitudinal fasciculus for Control > DP. Note that in Thomas et al., 2009, a threshold of an uncorrected p<0.01 followed by a cluster extent threshold of 10 voxels would represent a corrected threshold of p>0.99.

We also conducted the same comparisons in the whole-brain without a skeletonized mask (no threshold mean applied based on FA). This is not advisable due to partial volume effects but avoided arbitrary mean FA thresholds that may in fact remove regions in which a population has deficits. However, these comparisons demonstrate the greater difficulty in separating false from true differences due to the increased number of comparisons. For the unthresholded mask, Monte-Carlo simulations with the smoothness (FWHMx = 8.3 mm, FWHMy = 11.7 mm, FWHMz = 10.2 mm) and whole brain mask used demonstrated that at an initial threshold of p<0.005 uncorrected, a cluster extent threshold necessary for a corrected p<0.05 was 2064 voxels. No voxels anywhere in the brain reached this corrected threshold. The largest cluster to emerge from any comparison within the mask was a cluster of 1148 voxels which had a corrected p = 0.40 and was for AD in the Corpus Callosum for Control > DP.

Hence, with a whole brain search, if differences are local, it is difficult to separate false positives and false negatives from true positives and true negatives.

**Supplementary Figure Legends**

Supplementary Figure S1. ILF and IFOF: Deterministic tractography**:** Inter-individual variability in DP subjects

a-e. Mean values from DP participants are indicated by solid grey lines, and mean and standard error values from control participants are depicted by bar graphs. The 16 participants with DP are individually plotted with colored crosses. For ILF and IFOF and control callosal tracts, there were no statistically significant group differences (Figure 1, Table 1). Values for DP participants followed a normal distribution around a mean similar to that found in controls. Note that FA values in absolute terms for controls were similar to those found for controls in Thomas *et al.* (2009). However, absolute values for %voxels and %fibers were much lower for controls in our study. This is likely due to the fact that the whole brain was scanned here but only partial brain scans were collected in Thomas *et al.* (2009). In addition, superior scanning parameters support better tractography as a whole (Berman *et al.,* 2013) and hence, the relative numbers of quantifiable tracts across the whole brain would be expected to be greater in our study.

Supplementary Figure S2. ILF and IFOF: Deterministic and Probabilistic tractography with group mask**:** Inter-individual variability in DP subjects

a-b. Mean values from DP participants are indicated by solid grey lines, and mean and standard error values from control participants are depicted by bar graphs. The 16 participants with DP are individually plotted with colored crosses. For ILF and IFOF, there were no statistically significant group differences (Figure 2, Table 4 and 5). Values for DP participants followed a normal distribution around a mean similar to that found in controls.

Supplementary Figure S3. FFA fibers: Defined by face-specific functional regions of interest: Inter-individual variability in DP subjects

a-b. Mean values from DP participants are indicated by solid grey lines, and mean and standard error values from control participants are depicted by bar graphs. The 13 participants with DP are individually plotted with colored crosses. For whole bundle FFA fibers, there were no statistically significant group differences (Figure 3, Table 4) and a significant difference in right local WM in FFA fibers at p<0.05 (one-tailed). Values for DP participants followed a normal distribution.

Supplementary Figure S4: ILF and IFOF tracts and FFA fibers: Voxel-wise comparisons: Inter-individual variability in DP subjects

a. Mean values from DP participants are indicated by solid grey lines, and mean and standard error values from control participants are depicted by bar graphs. The 16 participants with DP are individually plotted with colored crosses. These RH and LH regions identified using voxel-wise comparisons were significantly lower in DP compared to controls. Values for DP participants followed a normal distribution. Note that for several DP subjects, values are below 0.2, which is the minimal threshold for tracking.

b. Voxel-wise comparisons within tracts and fibers of interest for MD: one region emerged past threshold for MD measures with DP > Controls. This RH cluster of 68 voxels was near the right local WM in FFA fibers. A cluster of 79 voxels was found in 63.7% of 10,000 random simulations at an uncorrected p<0.005, for a corrected p = 0.64. No regions emerged for Controls > DP.

d. Voxel-wise comparisons within tracts and fibers of interest for RD: one region emerged past threshold for RD measures with DP > Controls. This RH cluster of 94 voxels was near the right local WM in FFA fibers. A cluster of 94 voxels was found in 54.9% of 10,000 random simulations at an uncorrected p<0.005, for a corrected p = 0.55. No regions emerged for Controls > DP.

e. Voxel-wise comparisons within tracts and fibers of interest for AD: two regions emerged past threshold for AD measures with Controls > DP. One LH cluster of 53 voxels was immediately above left local WM in FFA fibers, and one RH cluster of 55 voxels was slightly anterior to right local WM in FFA fibers. A cluster of 53 voxels was found in 69.6% and a cluster of 55 voxels was found in 68.9% of 10,000 random simulations at an uncorrected p<0.005, for a corrected p = 0.70 and p = 0.69 respectively. No regions emerged for DP > Controls.

f. Voxel-wise comparisons within tracts and fibers of interest for FA: Two regions emerged past threshold for FA measures with Controls > DP and are described in the main text. For DP > Controls, one RH cluster of 146 voxels emerged and was located posterior to right local WM in FFA fibers. A cluster of 146 voxels was found in 41.8% of 10,000 random simulations at an uncorrected p<0.005, for a corrected p = 0.42.
