# Supplementary material for: Local but not long-range microstructural differences of the ventral temporal cortex in developmental prosopagnosia
Source: Neuropsychologia. 2015 Nov;78:195–206. doi: 10.1016/j.neuropsychologia.2015.10.010 (PMC4640146; doi:10.1016/j.neuropsychologia.2015.10.010)

ILF and IFOF: Deterministic and Probabilistic tractography with group masks (Interindividual variability in DP subjects)

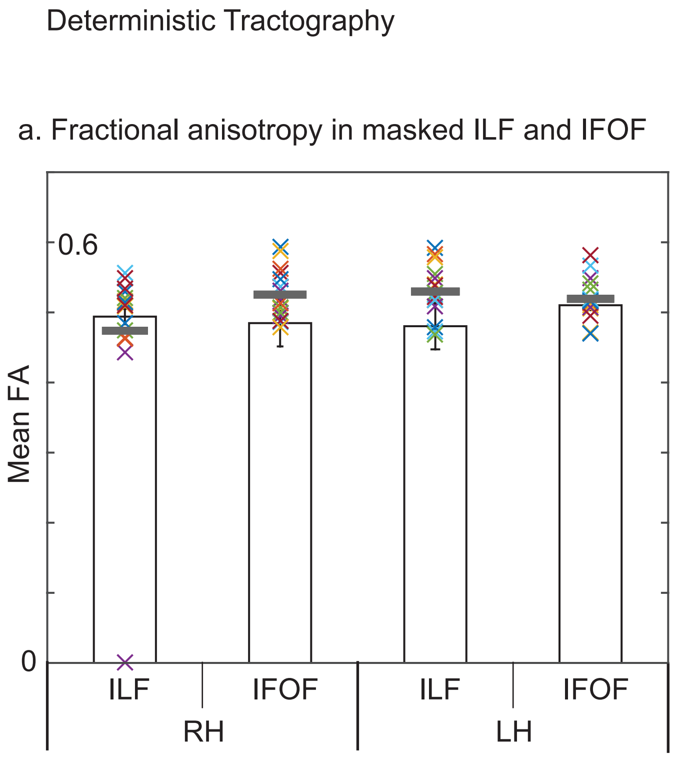

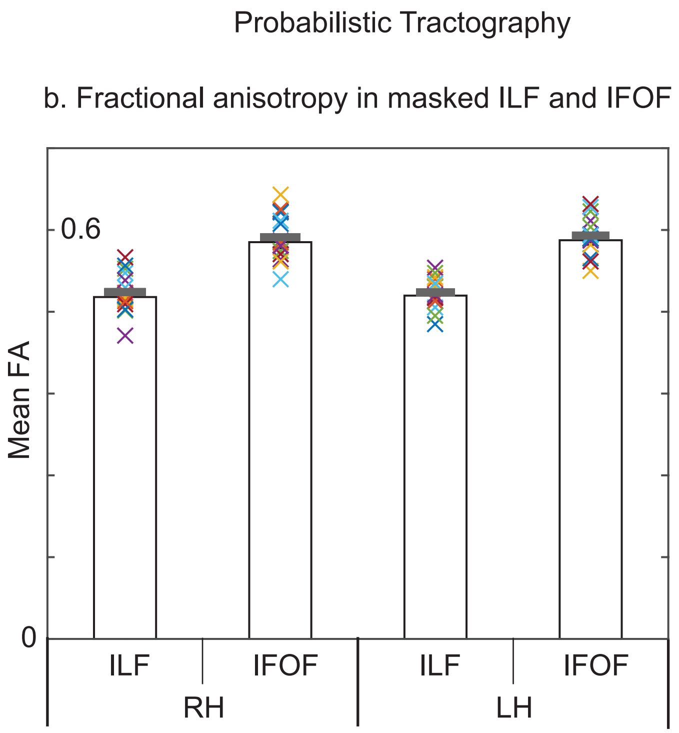

Supplement: Supplementary file 3 — Supplementary material [file mmc3.pdf]
