# Supplementary material for: Local but not long-range microstructural differences of the ventral temporal cortex in developmental prosopagnosia
Source: Neuropsychologia. 2015 Nov;78:195–206. doi: 10.1016/j.neuropsychologia.2015.10.010 (PMC4640146; doi:10.1016/j.neuropsychologia.2015.10.010)

FFA fibers: Defined by face-specific functional regions of interest
(Interindividual variability in DP subjects)

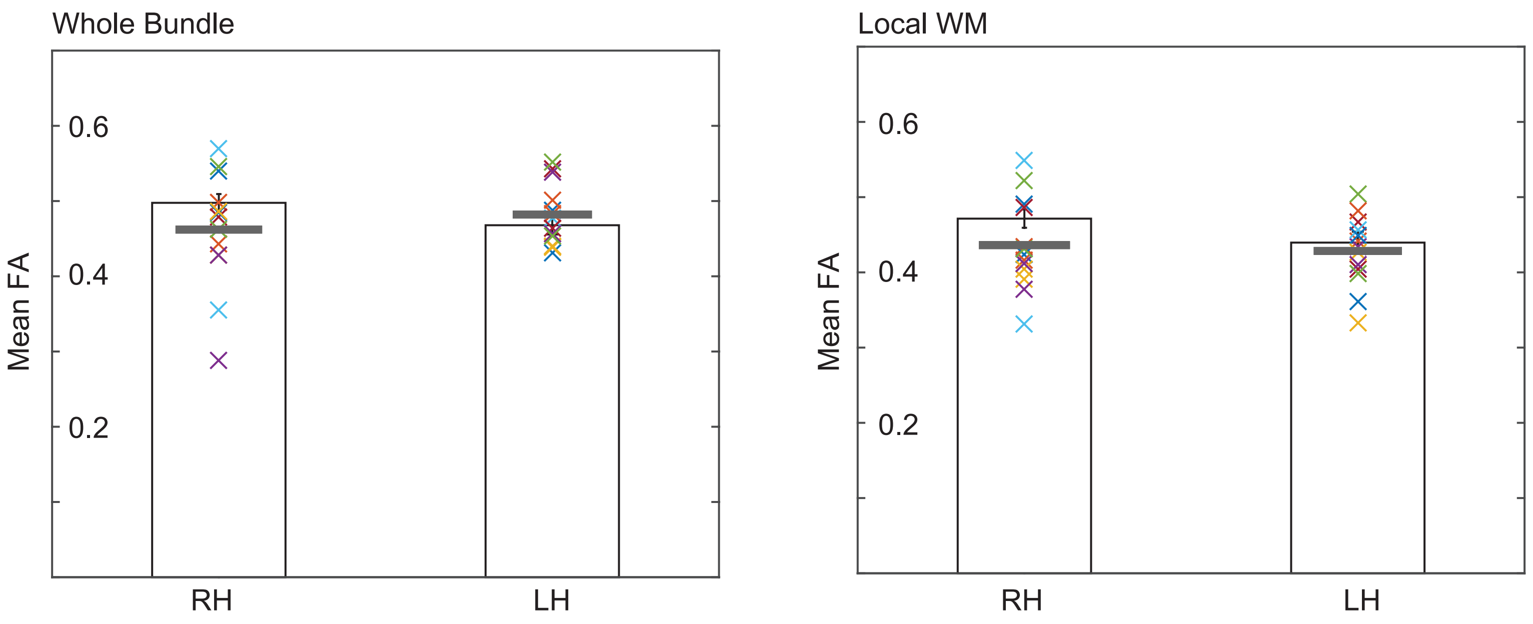

Supplement: Supplementary file 4 — Supplementary material [file mmc4.pdf]
