# Supplementary material for: Local but not long-range microstructural differences of the ventral temporal cortex in developmental prosopagnosia
Source: Neuropsychologia. 2015 Nov;78:195–206. doi: 10.1016/j.neuropsychologia.2015.10.010 (PMC4640146; doi:10.1016/j.neuropsychologia.2015.10.010)

ILF and IFOF tracts and FFA fibers: Voxel-wise comparisons within tracts and fibers of interest (Interindividual variability in DP subjects)

a. Fractional anisotropy in RH and LH clusters

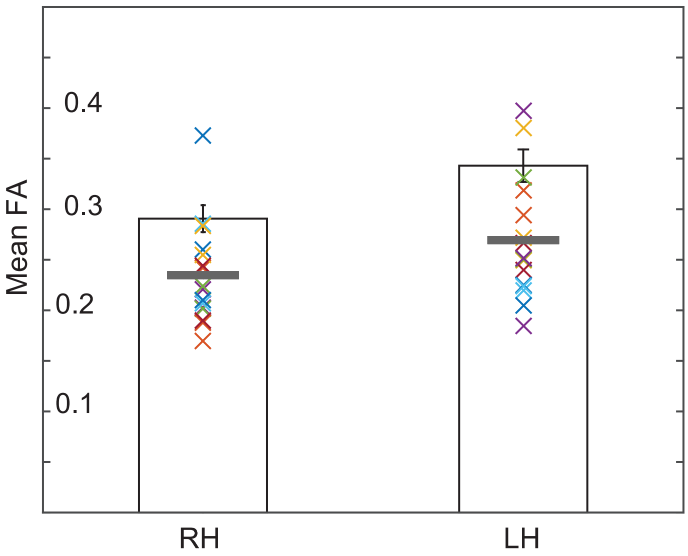

ILF and IFOF tracts and FFA fibers: Voxel-wise comparisons within tracts and fibers of interest (other metrics at p<0.005 uncorrected and greater than 40 voxels)

c.Mean Diffusivity (DP > controls)

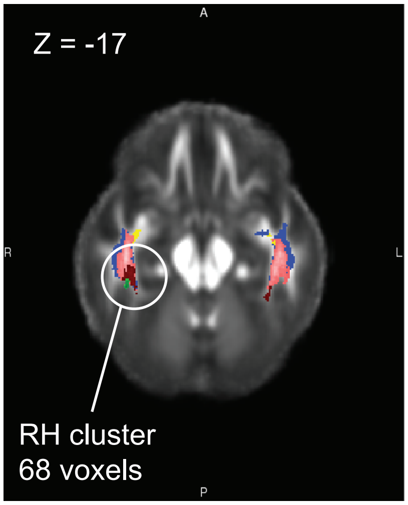

e. Axial Diffusivity (Controls > DP)

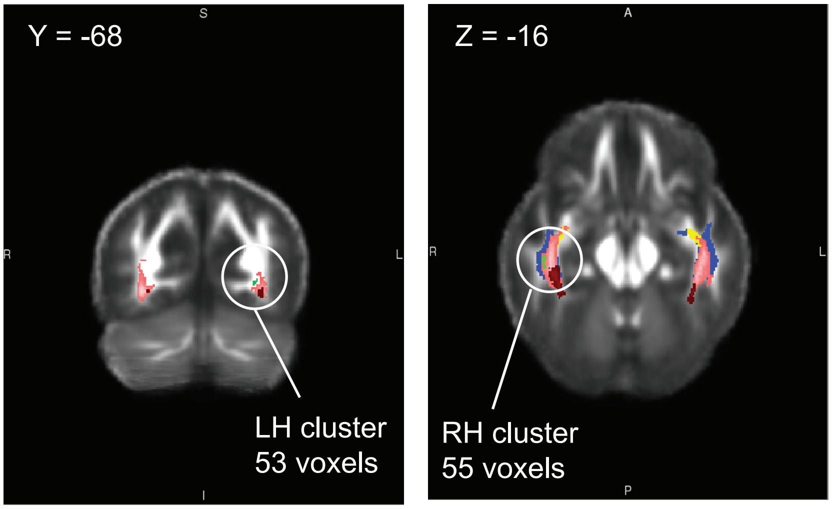

d. Radial Diffusivity (DP > controls)

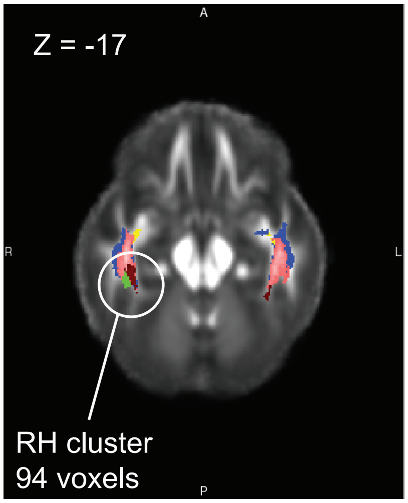

f. Fractional Anisotropy (DP > controls)

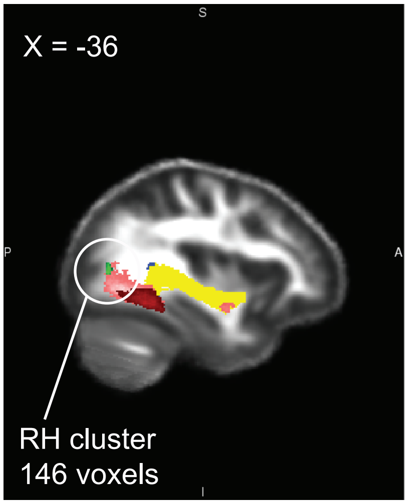

Supplement: Supplementary file 5 — Supplementary material [file mmc5.pdf]
